# A Review of the Effects of Raw Material Compositions and Steam Curing Regimes on the Performance and Microstructure of Precast Concrete

**DOI:** 10.3390/ma15082859

**Published:** 2022-04-13

**Authors:** Yucheng Zhou, Yijian Zhan, Mintao Zhu, Shengyi Wang, Juanhong Liu, Ning Ning

**Affiliations:** 1 Shanghai Construction Group Co., Ltd., Shanghai 200080, China; wangshengyi@scgtc.com.cn; 2 Shanghai Engineering Research Centre of Mega Structure High-Performance Concrete, Shanghai 201114, China; 3 Shanghai Construction Building Materials Technology Group Co., Ltd., Shanghai 200086, China; 4 College of Civil and Resource Engineering, University of Science and Technology Beijing, Beijing 100083, China; juanhong1966@hotmail.com; 5 NCO School, Space Engineering University, Beijing 102249, China; iris_ning1108@163.com

**Keywords:** precast concrete, steam curing, performance evolution, microstructure

## Abstract

In this paper, the effects of steam curing conditions on concrete properties and microstructural characteristics are reviewed, and technical approaches such as appropriate raw material compositions and curing regimes are explored. Moreover, the environmental effects of precast concrete are evaluated. The main conclusion is that steam curing can improve the early strength of concrete, but thermal damage, shrinkage cracking, delayed ettringite formation (DEF), and other factors cause the later strength to increase more slowly or even deteriorate. Accordingly, it is necessary to undertake methods for improvement: (1) Adopt a lot of high-activity mineral admixture + a few low-activity mineral admixture combinations to ensure that the early strength of concrete meets the standard while allowing the subsequent development of concrete hydration to ensure durability. (2) Control the precuring time and temperature gradient of the concrete to allow the initial structure of the concrete to form. (3) Use effective secondary curing, such as soaking in an aqueous solution of limestone, in addition to standard curing to further improve the compactness of concrete. Moreover, the replacement of cement with less than 30% mineral admixtures in steam-cured concrete should be promoted to alleviate the environmental hazards caused by excessive CO_2_ emissions.

## 1. Introduction

**Figure 1:**
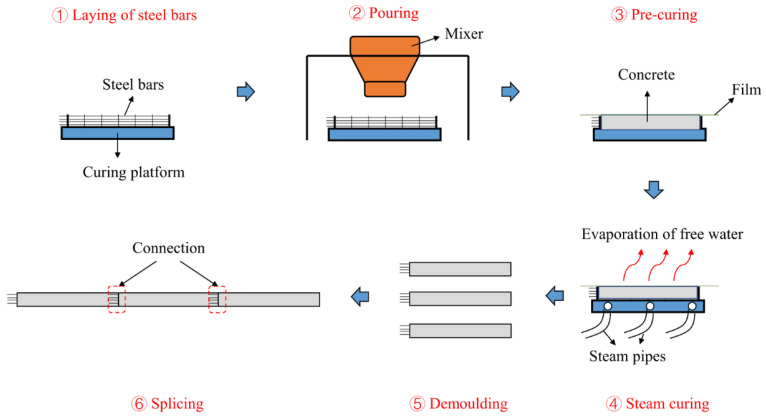
The production and application of PC.

With the rapid development and construction of national infrastructure, the demand for concrete is increasing. At this stage, concrete is mainly transported by truck and poured using pumping equipment. However, the pouring method has many drawbacks, such as pipe blockage, urban noise pollution, and insufficient strength of the concrete. As a result, precast concrete (PC) components have become widespread. Construction with PC components includes standardized building design and the production and molding of components in a factory. The components are transported to the construction site for assembly (Figure 1). The most widely used assembly method of PC components is wet connection, which adopts various connection techniques, such as grouting sleeves, lap joints, and 90° bending, to ensure the continuity of the PC structure [1]. PC structures have been widely used in residential, commercial, and industrial buildings [1]. The advantages of PC lie in its flexible and convenient production process, excellent product quality, and short construction period [2]. As a result, PC can greatly save time and cost at the construction site while ensuring the durability of concrete [3].

The properties of PC components highly depend on their raw material and production process. Currently, most PC component factories use cementitious materials based on ordinary Portland cement for high-strength concrete components, such as pipe piles. Some concrete with low strength grade requirements, such as wall panels, hanging panels, and pipe culverts, is blended with mineral admixtures to improve its durability. However, the partial replacement of cement with mineral admixtures also limits the compressive strength of PC, especially in the early stage. Moreover, the selection of aggregates is also very important for PC with high early strength demands. After casting, the PC components are cured in the factory. There are many methods for curing PC, for example, steam, dry heat, moist heat, microwave [4], and CO_2_ curing [5]. Compared with other curing methods, steam curing is the most widely used because of its advantages of high heat and humidity [6]. Steam curing promotes the development of concrete strength and improves production efficiency. However, if the steam curing temperature is too high or too low or if the curing time is too long or too short, the designed performance will not be achieved. Eventually, retrogression of the later strength and the appearance of fine shrinkage cracks in some PC components can be observed [7].

This paper compares the differences in properties and microstructure between PC made with pure cement and cement-mineral admixtures. Moreover, the effects of steam curing time, curing temperature, and secondary curing modes on PC are reviewed. In addition, the CO_2_ emissions of PC in the processing of raw material compositions and curing regimes are discussed. The corresponding work can provide a theoretical basis and technical support for the production and research of PC components.

## 2. Effect of Raw Material Compositions on PC

### 2.1. Pure Cement PC

#### 2.1.1. The Early-Stage Effect of PC

(1) Early strength: As shown in Table 1, the 1d compressive strength of pure cement concrete can be greatly improved by using the steam curing system instead of standard curing. The range of strength improvement in concrete is between 15% and approximately 92% [7,8,9]. Steam curing achieved a greater improvement in the compressive strength of low-strength concrete than that of high-strength concrete in the work of Vaasudevaa [7], while Zhang [8] and Gesoğlu [9] obtained the opposite results. Therefore, various factors, such as the curing process, should be considered relevant to the difference in the influence of steam curing on the mechanical properties of concrete.

(2) Early hydration reaction rate: The hydration rates of C_3_S, C_2_S, C_3_A, and C_4_AF in cement can be increased under high temperatures of steam curing, and the increase in C_2_S hydration is the smallest [10,11]. Studies have shown that the hydration reaction rate of cement is increased by a factor of approximately five with steam curing at 80 °C and by a factor of nine at 100 °C [6]. However, according to some studies, the curing temperature has a strong effect on the cement hydration rate when the curing temperature is below 60 °C, and the phenomenon is weakened when the curing temperature exceeds 60 °C [12]. Overall, steam curing can improve the reaction rate of the phases in concrete to a certain extent compared with curing at normal temperatures.

In a high-temperature environment, the rapid drying of the concrete surface and internal moisture make it difficult to continue hydration. While ensuring the temperature conditions required for concrete hardening, continuous humidification can slow the volatilization of water and promote the hydration of concrete. The increase in the chemical reaction in the concrete can generate more gel to fill the pores. The longer the time at high temperature, the more obvious the decrease in the concrete pores. This phenomenon plays an important role in the early strength of concrete [13].

(3) Early hydration products: Many studies have discussed the effect of high-temperature curing conditions on concrete hydration products. Bahafid [14] found that the density of C-S-H increased from 1.88 to 2.10 g/cm^3^, and Ca/Si decreased from 1.93 to 1.71 when the temperature in the curing environment was increased from 7 to 90 °C. Shen [15] used ^29^Si NMR (nuclear magnetic resonance) spectroscopy and nanoindentation techniques to study the effect of steam curing at 90 °C for 48 h on the nanoscale mechanical properties and microstructure of ultra-high-performance concrete (UHPC) and concluded that high-temperature steam curing could improve the density of C-S-H and promote structural shifts to the Q^3^ type. This transformation results in the formation of high-hardness product-defective tobermorite. Zhou [16] showed that a larger proportion of amorphous Al_2_O_3_ from mineral admixtures entered the C-S-H structure at high temperatures. The aluminum-oxygen tetrahedra replaced the bridged silicon-oxygen tetrahedra in the molecular chain, and the C-A-S-H structure formed.

**Figure 2:**
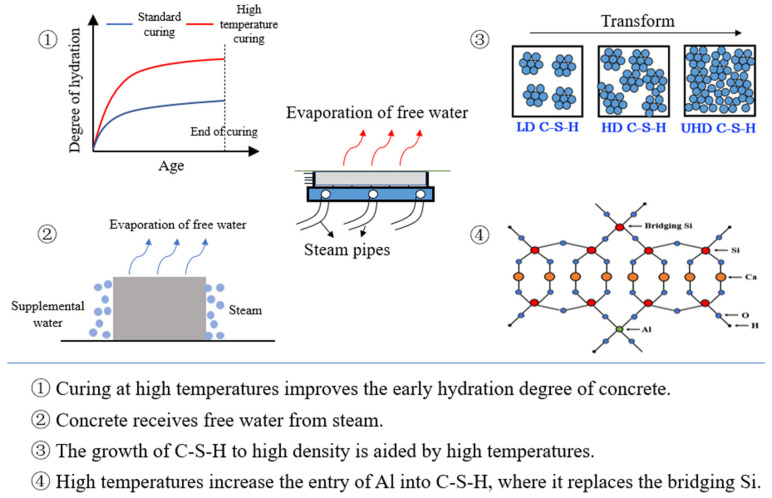
The effect of steam curing on the microstructure of concrete [16].

Overall, steam curing can not only promote the chemical reaction of concrete in a short time but also tends to transform C-S-H into a denser structure. Therefore, steam curing can improve the early performance of concrete (Figure 2).

#### 2.1.2. The Later-Stage Effect of PC

The high temperature of steam curing can accelerate the early hydration of cement and generate hydration products with high compactness. However, the final hydration degree of cement cured at a high temperature is lower than that obtained with standard curing [11,17]. Table 2 shows the difference in the post-cure strength of concrete subjected to steam and standard curing, as reported in the literature. In general, many scholars [7,8,9,18] have found that the performance of steam-cured concrete deteriorates obviously in the later stage, and the 28 d compressive strength of concrete can be reduced by up to 20%.

In addition, steam curing can cause many problems in concrete, such as thermal damage, shrinkage cracking, and delayed ettringite formation (DEF). These factors have a great impact on the subsequent performance of steam-cured concrete and can shorten its service life.

**Figure 3:**
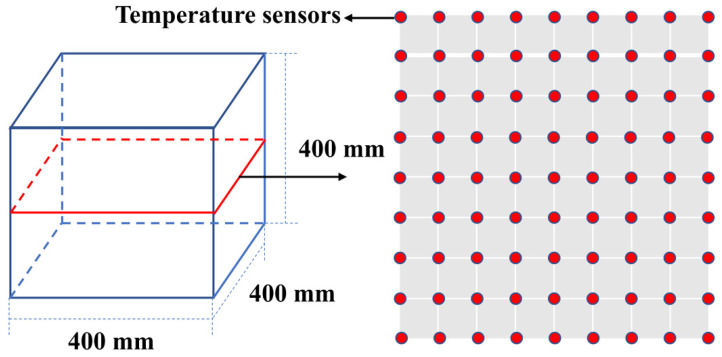
Thermal damage test of PC [20].

**Figure 4:**
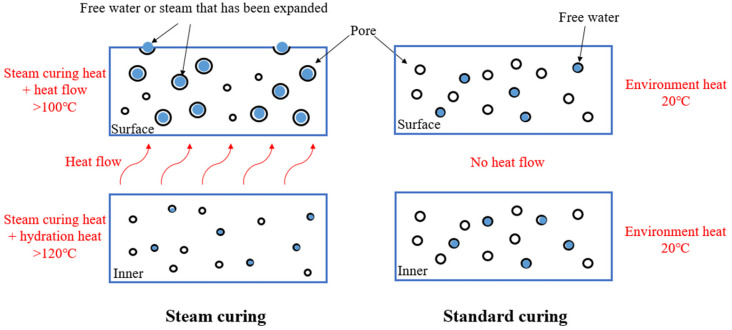
The heat damage of steam-cured concrete [20].

(1) Thermal damage: Some steam-cured concrete railway track slabs develop cracks after several years of service. The long-term fatigue load of high-speed trains promotes the coarsening of pores at the outer surface of the concrete due to thermal damage, and the pores expand to form large cracks. Corrosive mediums are then more likely to enter the interior of the concrete and cause a substantial decline in its performance [19]. Ma [20] designed an experiment to measure the temperature cloud inside concrete under the conditions of steam curing and standard curing (Figure 3). He found that the surface layer of concrete could reach temperatures greater than 100 °C under the combined action of hydration heat and high-temperature steam curing. A temperature of more than 100 °C expanded the surface water and steam, which caused irreversible deformation and swelling of the pores (Figure 4).

The temperature stress of the concrete surface intensifies and causes damage with increasing steam curing temperature. The temperature stress promotes the coarsening of the porosity of the outer layer (mainly large pores with a diameter in the range from 0.5 to approximately 4 mm) and the generation of cracks in the interface transition zones [21]. The higher the temperature and the longer the curing time, the more obvious the reduction in the strength of concrete in the later stage [22].

However, the thermal damage from steam-cured concrete is hardly reflected in normal testing, which is related to the size of the concrete specimen. The concrete used in the test is generally a 100 mm × 100 mm × 100 mm cube, its hydration heat is limited, and the surface temperature is not very high. Therefore, the early strength of steam-cured concrete is greatly improved, but the later strength does not decrease in some tests [23].

The secondary hydration of mineral admixtures with low activity is often used to compensate for the pores to effectively reduce the effect of thermal damage on concrete. Meanwhile, the steam curing process is controlled to alleviate the problem of excessive pore coarsening on the concrete surface.

(2) Shrinkage cracking: Slight volume deformation occurs in the normal curing process of concrete. The degree of change in the volume of concrete is correspondingly enlarged due to the improvement of various reaction rates in the steam curing environment. The higher the temperature is, the faster the deformation growth [24]. Wang [25] stated that pure cement concrete exhibits the phenomenon of “expansion-shrinkage” during the steam curing process. The concrete is not hardened enough to resist expansion caused by steam pressure during the heating process and shrinkage caused by the loss of water inside the concrete. Ninety percent of the free water in the concrete is distributed in pores smaller than 500 nm. Free water mainly evaporates in the precuring stage, and the free water content decreases less in this process than in other stages. However, the free water undergoes a substantial reduction due to the hydration reaction in the heating and constant temperature stages. Finally, the hydration rate slows, and the corresponding free water content decreases slowly in the cooling stage [26]. Similarly, Shen [27] found that the autogenous shrinkage of UHPC occurs mainly in the steam curing stage. This phenomenon is caused by the rapid consumption of water in the capillary pores and the increase in internal tension, and the autogenous shrinkage rate of UHPC is positively related to the fumed silica content. The expansion coefficient of concrete reached the approximate range of 15~20 × 10^–6^/°C in the heating stage, and the condensation of water vapor in the cooling process reduced the vapor pressure in the capillaries. The cracks occurred in the cooling stage because the concrete strength had not adequately developed to resist the shrinkage force [28,29].

The main control method for PC volume shrinkage is to add mineral admixtures and fibers. For example, the total volume deformation of concrete can be reduced by between 3% and 14% after adding 45% fly ash [25]. In addition, 1.5% fiber can reduce the shrinkage deformation of concrete by 50% [30]. Therefore, it is necessary to control the curing process and raw material compositions of steam-cured concrete to prevent deformation or even cracking of concrete.

**Figure 5:**
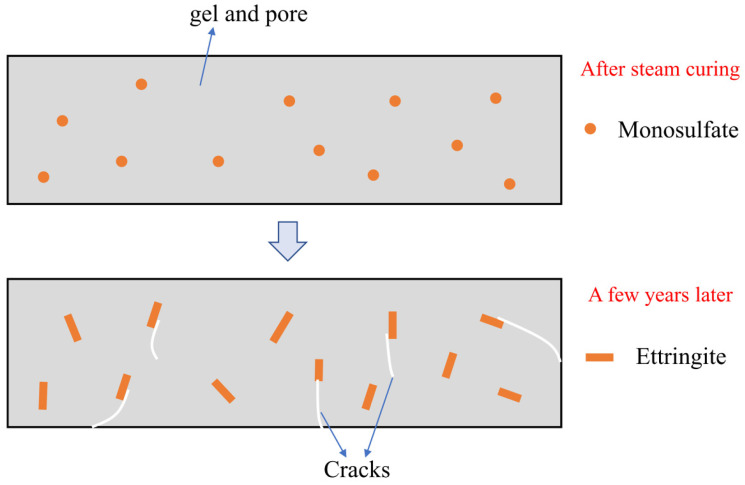
DEF damage.

(3) Delayed ettringite formation (DEF): It is generally believed that ettringite tends to be unstable above 70 °C, and it can be present in cement slurries up to 90 °C when the sulfate concentration is sufficient [31]. The formation of ettringite in the early stage is inhibited when the temperature exceeds 70 °C. Portions of the S and Al that are present in the hydration products are released later, resulting in DEF damage (Figure 5) [32].

In general, DEF content can characterize the impact of this failure on the concrete. Gu [33] proved that the thermal dissolution test (heating temperature 68 °C, duration 1 d) could detect the DEF content of concrete after curing at a high temperature (80 °C) without destroying the microstructure. Many factors affect the occurrence of DEF in high-temperature cured concrete. Ma [19] found that railway concrete with steam curing at temperatures less than 70 °C showed DEF damage after 4 years of service. Zhuang [34] reported that slightly increasing the curing temperature of steamed concrete exacerbates DEF damage, and mixing slag and fly ash in concrete can slightly decrease this damage. However, this improvement is much less than the negative effect of slightly increasing the steaming temperature. Zhang [35] found that DEF is strongly related to the molar ratio of SO_3_/Al_2_O_3_ in cement, and DEF damage may not occur at SO_3_/Al_2_O_3_ less than 0.8, while maximum expansion occurs at SO_3_/Al_2_O_3_ of approximately 1.1. The DEF can be related to other parameters of the cement. Escadeillas [36] used CaSO_4_ and Na_2_SO_4_ as additives, showing that high alkalinity of the pore solution in concrete is crucial for the occurrence of DEF and advocating that industrial wastewater containing Na_2_SO_4_ should not be recycled in high-temperature cured concrete.

Therefore, the steam curing temperature is a key factor affecting the DEF of concrete. The temperature during the steam curing process should not be too high; otherwise, the formation of ettringite will be inhibited. For the content of SO_3_ in the cement used for steam-cured concrete, the content of Na_2_SO_4_ in the mixing water should also be controlled to ensure the long-term performance of steam-cured concrete.

### 2.2. Cement-Mineral Admixtures PC

The mineral admixtures are generally fly ash and blast furnace slag powder. Some scholars have proposed the use of new types of solid waste powder. The specific research is described below.

#### 2.2.1. Cement-Fly Ash Type PC

Fly ash contains approximately 35% crystalline mullite and 60% amorphous phase, according to the results of X-ray diffraction (XRD)-Rietiveld [37]. Although the amorphous phase content of fly ash is relatively low, its activity level is not reflected in the amorphous phase content [38]. Compared with blast furnace slag powder, fly ash has a larger [SiO_4_]^4−^ tetrahedral polymerization degree in its amorphous phase and even a three-dimensional network structure. The [SiO_4_]^4−^ structure with a large degree of polymerization is not easily excited by Ca(OH)_2_ [39]. Therefore, fly ash belongs to the class of mineral admixtures with low activity.

In the normal temperature curing process, the addition of fly ash causes dilution of cement, which can promote cement hydration to a small extent [40], but fly ash reacts very little during the early stage. Studies have shown that in concrete curing at 7, 23, and 50 °C, the hydration of fly ash starts at 90, 7, and 1 d, respectively, and high temperature can accelerate the reaction of amorphous SiO_2_ and Al_2_O_3_ from fly ash with Ca(OH)_2_ to form new products [41]. When the curing temperature reaches 50 °C, or fly ash has a high substitution rate, fly ash competes with cement to adsorb free water, thus hindering the hydration of cement [42,43]. Yazici [44] found that as the fly ash content increased from 0 to 70%, the 1 d strength of steam-cured concrete decreased continuously until it was only 40% of that of pure cement concrete. However, steam curing can increase the 1 d strength of 30% fly ash + 70% cement concrete by 15% to 44% [7]. Both Yazici [44] and Wang [25] believed that although the addition of fly ash would reduce the early strength of steam-cured concrete, it could also make the pore size distribution of concrete more reasonable, reducing the total shrinkage. Additionally, the effective diffusion coefficient of C-S-H around the cement particles increases after the incorporation of fly ash, which promotes the diffusion of water through the C-S-H layer and thus contributes to the hydration of concrete in the later process [43], providing excellent durability of cement–fly ash steam-cured concrete.

Due to the extremely stringent requirements for the early strength of PC, the applicability of fly ash is weak. Some scholars have attempted to improve it to meet the requirements of practical applications. Yang [45] ground fly ash into tiny particles with an average diameter of 2.29 μm to stimulate its early activity. The experiment found that ultrafine fly ash enhanced the pozzolanic reaction degree of concrete and increased the C-(A)-S-H Al/Si and average main chain length. When the content of ultrafine fly ash did not exceed 30%, the early and late strengths of steam-cured concrete were better than those of pure cement concrete. Zou [46] promoted the hydration of cement to generate more Ca(OH)_2_ to react with fly ash by adding triisopropanolamine and found that a dosage of 0.1% could improve the early compressive strength of cement-fly ash PC by 20%. Liu [47] showed that the incorporation of nano-SiO_2_ can promote the hydration and pozzolanic reaction of cement and fly ash, fill pores, and optimize the pore structure, and 4% nano-SiO_2_ can double the compressive strength of steam-cured mortar. Mei [48,49] found through experiments that Na_2_SO_4_ can promote the hydration of fly ash, and the synergistic effect of Na_2_SO_4_ and nano-SiO_2_ on the mechanical properties and microstructure of steam curing cement-based materials is particularly obvious.

Because the activity of fly ash is very low, it is almost unsuitable for mixing in steam-cured concrete. The early mechanical properties of cement-fly ash PC can be considerably improved by means of physical and chemical excitation, but there is a lack of analysis of the corresponding feasibility of practical production and limited research on durability characteristics at this stage.

#### 2.2.2. Cement-Blast Furnace Slag Powder Type PC

Approximately 80% of blast furnace slag powder is amorphous and easily activated in the cement system [39]; thus, blast furnace slag powder is a kind of mineral admixture with high activity. Blast furnace slag powder also has a high sensitivity to curing temperature, and the higher the temperature is, the more obvious the early strength gain effect of slag concrete [50].

When cement-slag concrete is cured at high temperatures, its early hydration speed is greatly increased, and its strength is much higher than that of concrete with the same replacement rate and standard curing, but its later performance is inferior to that of room temperature-cured concrete [51]. Compared with pure cement concrete, the early compressive strength of cement-slag concrete is decreased to a certain extent after steam curing. For example, Castellano [50] found that partially replacing cement with slag considerably reduced the early performance of steam-cured concrete, and the higher the replacement rate was, the more obvious the loss of strength. Nguyen [52] reported that the early strength of 50% cement + 50% slag steam-cured concrete was slightly lower than that of pure cement concrete, and the strength was similar after adding a small amount of gypsum. However, steam curing damages the later strength of cement-slag concrete. Several studies show that the early compressive strength is the same for 50% cement + 50% slag steam-cured concrete and pure cement steam-cured concrete, and the 28 d strength decreases from 10 to 20% [53]. This difference is most likely caused by factors such as the specific surface of the slag, but in general, the incorporation of slag will affect the early performance of steam-cured concrete to a certain extent.

The addition of slag can reduce the content of C_3_A and C_4_AF in the cementitious system, thereby improving the corrosion resistance of steam-cured concrete. Yan [54] studied the effect of 20, 50, and 70% slag substitution on the sulfate corrosion resistance of steam-cured concrete. The results showed that the higher the slag content is, the lower the ettringite XRD peak; steam-cured concrete with 20 and 50% slag content is damaged due to the crystallization pressure of ettringite and gypsum, while 70% slag can form aluminum-containing hydration products, reducing the risk of expansion and damage due to the formation of ettringite. Yan [55] added between 0.5 and approximately 1.0% ethylenediaminetetraacetic acid to steam-cured concrete to complex Ca(OH)_2_ to retard the production of expansion products, which solved the problem of low-volume slag steam-cured concrete being affected by sulfate corrosion.

The addition of blast furnace slag powder slightly reduces the early and late mechanical properties of steam-cured concrete, but it improves the corrosion resistance. To ensure the full-cycle performance of steam-cured concrete and alleviate the problems of high cement prices and environmental pollution, the slag contents of high-strength and low-strength grade PC are often kept below 20% and 40%, respectively, in industrial production.

#### 2.2.3. Cement-Other Admixture Type PC

In the current research, in addition to the most common use of fly ash and blast furnace slag powder, some scholars have explored materials such as limestone powder and metakaolin to replace cement and prepare PC through steam curing.

(1) Limestone powder: Limestone powder has two kinds of crystal structures, namely, calcite and aragonite. Both crystal structures are relatively stable in the environment below 600 °C and serve as inert admixtures in concrete. Calvo [18] used 20% limestone powder to replace cement, and according to the Fuller curve, the densest stacking skeleton was achieved, indicating that limestone powder with a low substitution rate had little effect on the strength of PC. The total porosity of concrete increased by between 1 and approximately 2%, but the pore size was biased towards small sizes, which can result in better durability performance than larger sizes. Panesar [56] found that 15% limestone powder can improve the early strength of steam-cured concrete without affecting the later performance. Moreover, the smaller the particle size of limestone powder, the better the performance of the concrete. Zhang [57] reported that when the replacement rate of limestone powder reaches 40%, it causes problems such as a reduction in the early strength of steam-cured concrete and increases in chloride ion permeability and carbonization depth.

(2) Metakaolin: Metakaolin is obtained by calcining and dehydrating kaolinite clay at 800 °C. Metakaolin is similar to fumed silica, with a high specific surface area and pozzolanic activity, and is therefore widely used [58]. Cassagnabère [59] used 25% metakaolin to replace cement in steam-cured concrete and found that its 1 d strength was approximately 20% higher than that of pure cement concrete, while the 28 d strength remained similar. This is due to the pozzolanic reaction of metakaolin in the steam-cured environment to generate C-(A)-S-H. Shen [60] believed that the optimal content of metakaolin is 10%, which can react with a large amount of Ca(OH)_2_ in concrete to generate ettringite, C_4_AH_13_, and C_3_ASH_6_ to offset the negative effects of thermal damage and shrinkage. Similarly, Mo [58] found through experiments that the optimal content of metakaolin in steam-cured concrete is 10–15%, and its 28 d strength is similar to that of standard-cured concrete with no obvious reduction.

**Figure 6:**
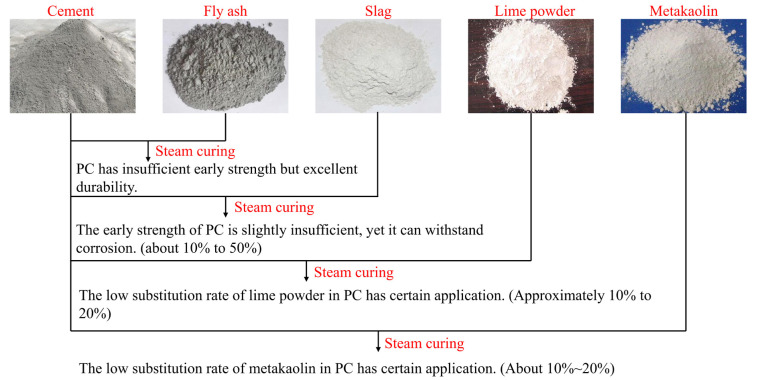
The application of mineral admixtures.

It is feasible to use admixtures such as limestone powder and metakaolin in steam-cured concrete (Figure 6), but further consideration should be given to factors such as cost and long-term stability.

#### 2.2.4. Cement-New Solid Waste Powder Type PC

In the past 100 years, the rapid development of industrialization has caused significant environmental pollution. The accumulation of industrial waste, tailings, and other wastes not only occupies a large area of land but also causes many environmental problems, such as water pollution and dust [61,62]. Many scholars have begun to study the use of new solid waste powder to replace cement, followed by steam curing to prepare PC.

**Figure 7:**
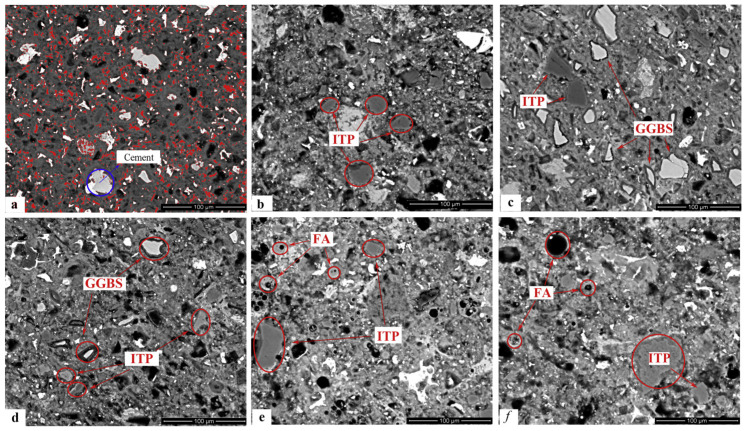
Backscattered secondary electron (BSE) images of steam-cured hardened pastes with iron tailing powder at 90 days: (**a**) 100% cement, (**b**) 50% cement + 50% iron tailing powder, (**c**) 50% cement + 15% iron tailing powder + 35% slag, (**d**) 50% cement + 35% iron tailing powder, (**e**) 50% cement + 15% iron tailing powder + 35% fly ash, and (**f**) 50% cement +35% iron tailing powder + 15% fly ash [67].

(1) Rice husk ash: Rice husk ash is a kind of residue formed after biomass fuel power plants use chaff as fuel. Vigneshwari [63] used ground rice husk ash to replace silica fume. Under the conditions of steam curing and a 40% replacement rate, its 28 d compressive strength was increased by 64% compared with that of cement-silica fume concrete, mainly because of rice husk ash. Steam curing enhances the pozzolanic reaction and thereby makes the concrete microstructure denser.

(2) Coal gangue: Coal gangue is a solid waste discharged from the coal mining process and coal washing process. Zhang [8] found that high-temperature (60 °C) steam curing can activate the activity of coal gangue and achieve an effect similar to the 1 d and 90 d strength of pure cement concrete, but too high a temperature (80 °C) will increase the internal defects of coal gangue concrete, which is not conducive to long-term performance, so there are certain disadvantages in the application of coal gangue in steam-cured concrete.

(3) Lithium slag: Lithium carbonate can be obtained by calcining lithium ore to 1200 °C, which produces industrial wastes such as lithium slag and some oxides. The main minerals in lithium slag are quartz (SiO_2_), spodumene, and dihydrate gypsum (CaSO_4_·2H_2_O). Li [64] stated that the addition of 20% lithium slag reduces the 28 d strength of steam-cured concrete, but improves its corrosion resistance. Simultaneously, steam curing can solve the problem of DEF damage due to the presence of SO_3_ in lithium slag.

(4) Nickel-iron slag: Nickel-iron slag is an industrial waste slag produced in the process of smelting laterite ore to produce nickel-iron alloy in an electric arc furnace at temperatures from 1500 to 1600 °C. Li [65] concluded that the addition of 20% nickel-iron slag would slightly reduce the 28 d strength of steam-cured concrete and would be basically the same at 240 d. Steam curing can promote the formation of hemicarbonate and C-A-S-H gels from Al in cement and nickel-iron slag, reducing the risk of sulfate attack.

(5) Palm oil fuel ash: Palm oil fuel ash is a product of palm oil fuel. Zeyad [66] used 20, 40, and 60% palm oil fuel ash to replace cement in standard cured concrete and found that its 1 d strength decreased by 28.7, 42.5, and 58.8%, respectively. There was no obvious decrease in the early strength of concrete after curing, mainly because steam curing can accelerate the pozzolanic reaction of palm oil fuel ash and increase the content of C-(A)-S-H in concrete.

(6) Iron tailing powder: Iron tailings are wastes that are left after iron ore beneficiation. Han [67] showed that the release strength of steam-cured concrete compounded with iron tailing powder (15%) and slag (35%) increased by 40% compared with that of pure cement concrete, and the later strength developed well. Both Han [67] and Cai [68] concluded that iron tailings tend to be inert materials at room temperature, but partial secondary hydration can occur under steam curing conditions, which can improve the performance of concrete to a certain extent (Figure 7).

Comprehensive utilization of solid waste and recycling of garbage are common goals worldwide. However, when various types of waste are applied to PC fabrication, full consideration should be given to the influence of Fe, Ni, and other trace elements on the long-term stability of concrete.

### 2.3. Special Aggregate Type PC

Considering the requirements of further alleviating waste storage or preparing special concrete, some studies research the use of construction waste concrete, light aggregate, and discarded PC to replace the commonly used crushed limestone in the preparation of PC.

(1) Recycled aggregate of construction waste concrete: Due to factors such as large porosity, strong water absorption, and rough and angular surfaces, the incorporation of recycled coarse aggregate increases the demand for high-performance water reducers to reduce the fluidity of concrete [69]. The increase in the replacement rate of recycled coarse aggregate obviously has an adverse effect on the early compressive and split tensile strengths of steam-cured concrete. However, the water stored in the pores of recycled coarse aggregate can achieve a long-term internal curing effect on steam-cured concrete and solve the problem of the effect of shrinkage of the strength [70]. In addition, Hanif [71] reported that the use of recycled coarse aggregate could reduce the shrinkage deformation of pure cement concrete by 15%, which can decrease the early cracking of concrete and improve its resistance to harmful ions. Gonzalez-Corominas [72] demonstrated that steam curing could compensate for the high porosity of recycled coarse aggregate concrete. Yammine [73] found that the concrete porosity caused by recycled coarse aggregate and recycled fine aggregate can counteract the expansion of part of the DEF and reduce the risk of failure.

(2) Lightweight aggregate: To reduce the dead weight of the structure, lessen the amount of materials required, and improve the efficiency of component transportation and hoisting, light aggregate PC can be used. Commonly used light aggregates include ceramsite and perlite. Long [74] indicated that replacing ordinary aggregates with lightweight aggregates would reduce the mechanical properties of steam-cured concrete. However, steam curing can improve the 1 d strength of 100% lightweight aggregate concrete to 21.3 MPa, which fully satisfies the requirements of the lightweight aggregate concrete standard. Water can be stored in the interior for later internal curing, so the strength of the two curing regimes is similar at 56 d [9]. Ogawa [75] applied porous ceramic fragments to the cement-fly ash steam-cured concrete, and such fragments can also play the role of internal curing to improve the mechanical and durability performance of concrete.

It is feasible to use construction waste to recycle coarse aggregates and light aggregates to prepare steam-cured concrete. However, the current stockpile of construction waste is low, and the demand for lightweight PC is also low. There is little research on this niche technology.

**Figure 8:**
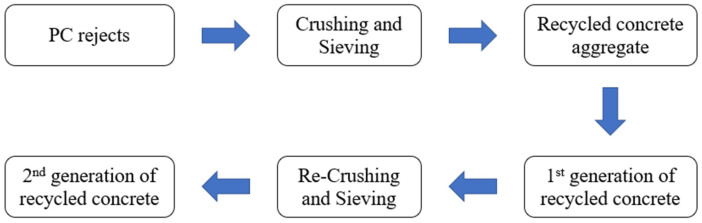
Evolution process of recycled PC [76].

(3) Recycling of discarded PC: Discarded PC, which is similar to ordinary building concrete, can also be used as recycled coarse aggregate for effective recycling (Figure 8) [76]. However, recycled PC also alters changes in concrete properties. Salesa [76] and Erdem [77] found that the mechanical properties of PC recycled aggregate are better than those of ordinary natural aggregate, and the 28 d strength of the corresponding recycled aggregate concrete can be increased by approximately 5% compared with ordinary aggregate concrete, but this improvement decreases as the number of recycled aggregate cycles increases. Soares [78] emphasized that the use of recycled PC aggregates and superplasticizers can greatly enhance the mechanical properties and durability of concrete. Recycling PC in a full life cycle management operation mode is a necessary approach in contemporary times.

### 2.4. CO_2_ Emissions from PC Raw Material

Since the 21st century, the rapid development of the world’s infrastructure, economy, and energy has also resulted in excessive greenhouse gas (CO_2_-based) emissions, and global warming has led to environmental problems such as melting glaciers and extreme climate events [79]. During the production of Portland cement (calcined limestone), a large amount of CO_2_ gas is released, accounting for between 5 and approximately 7% of total CO_2_ emissions. According to the Global Climate Summit, to achieve the goal of controlling the global temperature rise to less than 2 °C by the end of this century and maintaining it within 1.5 °C, the production of cement should be limited by 2030 to achieve the goals of the Paris Agreement and must be decreased by at least 16%. To alleviate the environmental problems caused by cement, it is imperative to promote the use of fly ash, mineral powder, and other industrial wastes in the concrete industry to replace most of the cement.

Table 3 shows that the production of 1 t of cement emits approximately 833–944 kg of CO_2_, accompanied by harmful gases such as SO_2_, CO, and NO_x_. The byproducts of industrial production, such as fly ash and blast furnace slag powder, are industrial solid waste residues and produce very little harmful gas [53,80,81]. The production of 1 kg of fine aggregate emits approximately 0.0013 kg of CO_2_, and 1 kg of coarse aggregate emits approximately 0.004 kg of CO_2_. However, many prefabricated component companies still use cement as the main cementing material, which not only presents economic problems but also causes excessive CO_2_ emissions. If 50% of cement can be replaced by mineral admixtures and the quality of components at all ages can be ensured, then the CO_2_ emissions generated by components can be reduced by approximately 30%. Furthermore, replacing ordinary aggregate with recycled coarse aggregate can reduce CO_2_ emissions by 2% [71]. This change is extremely important and meaningful for alleviating the environmental problems of global warming.

### 2.5. Summary and Prospects

High cement content PC has the advantage of high early strength, but it will also increase greenhouse gas emissions such as CO_2_ and produce concrete with insufficient durability. Replacing a portion of cement with fly ash or slag can alleviate durability problems such as thermal damage, shrinkage cracking, and DEF. Although a large number of mineral admixtures used in the place of cement can reduce greenhouse gas emissions, they also cause decreases in early strength. Therefore, it is urgent to coordinate the early strength, durability, and environmental benefits of PC. There is still great uncertainty in the application of new solid waste powder and recycled aggregate, and a large number of durability tests are needed to verify the quality of PC before it can be used in engineering applications.

## 3. Effect of Steam Curing Regimes on PC

The curing process is one of the most important factors affecting the mechanical and durability performance of steam-cured concrete. The steam curing of concrete can be divided into four stages: precuring, heating, constant temperature, and cooling [37]. In addition, when steam curing is completed, some component plants adopt secondary curing methods, such as autoclaves, to further improve the early strength of the PC to ensure its suitability for engineering applications.

### 3.1. Effect of Temperature Gradient on PC

**Figure 9:**
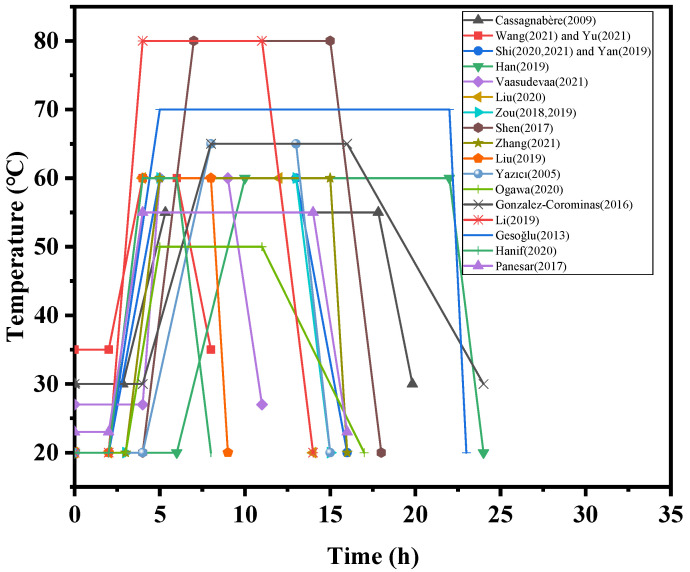
Treatment time of steam-cured concrete at different stages [7,8,9,21,25,28,29,44,47,54,56,59,60,65,67,72,73,75,82,83,84,85,86,87].

Figure 9 shows the steam curing stages used to study the effect of steam curing on concrete reported in the literature. The entire curing process was maintained for 24 h, and the maximum temperature was 80 °C.

The first stage is the static curing stage of concrete (between 2 and approximately 6 h), in which the mixed concrete is loaded into the mold and usually covered with a plastic film to maintain moisture. Shi [84] found that when the concrete was covered during the steam curing process, a layer of film could reduce the connected pores formed by the exchange of moisture and heat, thereby decreasing thermal damage; geotextile is more effective than plastic film. During the precuring process, the concrete begins to harden, and the initial structure continues to form. The precuring time is related to the cementitious material components; the precuring time of pure cement concrete is 6 h, which is the most suitable [88]. Moreover, prolonging the resting time (from 2 to 8 h) and the smoothing process before heating can also reduce thermal damage [85]. Thus, allowing sufficient precuring time can provide space for initial structure formation in concrete and reduce the risk of thermal damage to concrete due to uneven settlement of hydration products during steam curing. According to Erdem [89], the positive effect of steam-cured concrete is greatest when the precuring time coincides with the setting time of the concrete.

The second stage is the heating stage of concrete (from 1 to approximately 3 h). The curing mold table is primarily heated at this stage until it achieves the highest steam curing temperature. Shi [83] investigated the influence of different heating rates on the characteristics of concrete and discovered that increasing the temperature from room temperature to 30 °C for 1 h, followed by heating to 60 °C at a rate of 30 °C/h, was beneficial for the first stage of concrete. The formation of the structure has the greatest benefit for the 90 d strength. To limit the thermal damaging effect of concrete, it is necessary to use a more moderate temperature increase during the heating stage.

The third step is the constant temperature stage of concrete (from 2 to approximately 10 h), which is the main curing stage of concrete. The curing mold table maintains a steady high temperature and adequate water vapor to enable full hydration of the concrete and enhance the early strength. Zdeb [88] concluded that increasing the steam curing constant temperature by 10 °C increased the early compressive and flexural strengths of concrete by 16 and 0.7 MPa, respectively. When the temperature exceeds 160 °C, tobermorite crystals form and fill the pores of the concrete, improving its mechanical properties [90]. However, the more obviously the early strength is increased, the more severely the later strength will weaken [91]. Due to energy usage and other considerations, the constant temperature in actual production processes is normally maintained between 50 and 80 °C. Zdeb [88] discovered that a constant temperature process lasting more than 12 h has no discernible influence on the mechanical properties of concrete and that a constant temperature of 6 h is the most appropriate. The curing period of concrete in a high-temperature environment has no positive influence on its qualities but instead reduces its later strength [90]. Duan [22] hypothesized that the duration of steam curing at temperatures of 40, 50, and 60 °C should be controlled between 6.2 and approximately 31 h, between 4 and approximately 19 h, and between 2.7 and approximately 13 h, respectively, which could balance the hydration degree of cement and the C-S-H rate. The microstructure formed by stacking achieves the desired performance.

The fourth stage is the cooling stage of the concrete (between 1 and approximately 3 h), which corresponds to the heating process. To reduce the risk of shrinkage cracking of the concrete during this process, it is necessary to control the rate of temperature decrease.

Therefore, it is necessary to control the curing time and temperature of each stage to ensure the early mechanical properties and long-term durability of steam-cured concrete.

### 3.2. Effect of Secondary Curing on PC

**Figure 10:**
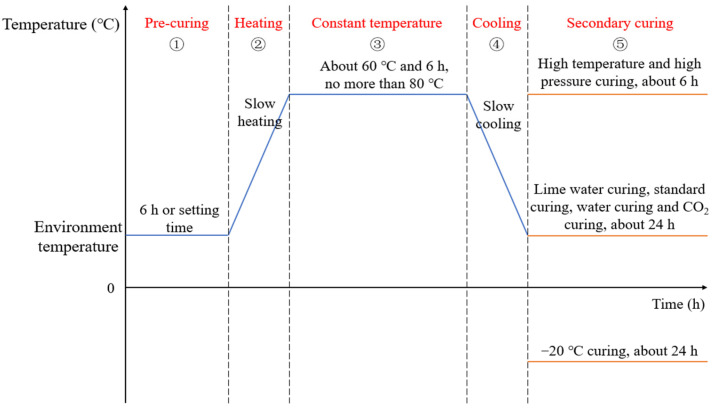
The curing regimes of PC.

To further strengthen the early mechanical properties and durability of steam-cured concrete, effective secondary curing is often carried out on the concrete after demolding (Figure 10).

Some milder curing methods, such as standard curing and water curing, can restore the chemical reaction degree of concrete and improve its compactness. Kang [92] added a 24 h 20 °C water curing process in the middle of the 48 h steam curing process, which added water to the concrete and improved its hydration and pozzolanic reaction. It was found that such a secondary curing process can significantly alleviate the thermal damage caused by high temperature, and the improvement is more obvious below 120 °C. Similarly, Li [93] found that after steam curing (90 ± 5 °C) for 6.5 h + high temperature and high pressure (180 ± 5 °C, 1.0 ± 0.05 MPa) for 6 h, water curing for 7 d alleviated the coarsening of concrete pores and improved the carbonization resistance and resistance to chloride ion penetration. Rostami [94] used carbonization curing for concrete after steam curing for 2 h, and the carbonization of Ca(OH)_2_ promoted the microscale densification of concrete and improved its durability.

Furthermore, some scholars have compared the effects of different types of secondary curing techniques on the performance of steam-cured concrete. Zou [87] conducted secondary curing tests with water at room temperature, 60 °C dry heat, −20 °C temperature, and standard conditions. The permeability of the steam-cured concrete surface increased after secondary curing with water at room temperature and with 60 °C dry heat. The negative temperature and standard curing conditions had a positive effect on the performance of PC, and the steam-cured concrete reached the same compressive strength as the standard-cured concrete at 28 d. In addition, Liu [86] further compared the follow-up effects of four secondary curing methods: an aqueous solution of lime, room temperature water, standard condition, and a natural air environment on steam-cured concrete and found that the subsequent strength of concrete under natural air conditions increased slowly due to the lack of water. However, long-term immersion in water can easily lead to the loss of hydration products; the improvement in the mechanical performance and durability of concrete were the most obvious after curing with a saturated aqueous solution of lime, followed by standard curing conditions. Yim [95] conducted in-depth research on standard curing conditions and tested secondary curing methods of 20 °C at 10% RH, 20 °C at 60% RH, and 20 °C at 90% RH for 7 d, and the results showed that CaO in concrete under high humidity reacted with C_2_S and was rehydrated to form hydration products such as CaCO_3_, C-S-H, and ettringite. The new products filled the microcracks and defects left by thermal damage, improving the performance of concrete.

Therefore, long-term standard curing and limestone water curing have the greatest advantages in improving the performance of PC. Effective secondary curing of PC can be carried out according to different performance and project duration requirements.

### 3.3. CO_2_ Emissions from PC Production

PC does not produce CO_2_ emissions in the precuring and cooling stages, but a large amount of CO_2_ is produced in the heating and constant temperature stages. PC usually uses a boiler for steam curing, and the oil used in the boiler emits 2.50 kg CO_2_/L; 10 L of boiler oil is consumed per hour [96]. Therefore, 1 m^3^ of concrete emits approximately 25 kg of CO_2_ in 1 h [53], and the CO_2_ emission of PC in the production process is positively correlated with the total time of heating and constant temperature. Overall, the heating time is approximately 3 h, and the constant temperature time is approximately 6 h, which can ensure the performance of PC and minimize CO_2_ emissions. Additionally, there is a certain amount of CO_2_ emissions in engineering applications such as transportation and construction [97]. The CO_2_ emissions in this process are too complex for the scope of this paper and can be researched in detail in the future.

### 3.4. Summary and Prospects

After long-term research and practice, the steam curing regimes of prefabricated component plants have been largely perfected. However, the maximum steam curing temperature, duration, and other parameters of the curing process need to be researched in detail by balancing multiple angles to finally achieve the maximum comprehensive effect of energy and performance. Moreover, to further reduce CO_2_ emissions and save energy, nonsteam PC can be researched.

The secondary curing methods in the existing research all require excessively long curing times, and the major advantage of PC lies in the rapid production of finished products and application in engineering. Therefore, in practical applications, it is necessary to improve the existing secondary curing methods and effectively replenish the concrete during long-distance transportation of PC components or sprinkle water and apply a film after the splicing and assembly are completed to ensure the performance of the concrete.

## 4. Conclusions

This paper summarizes the effect of steam curing on concrete properties, the improvements in performance achieved by different raw material combinations and curing regimes for concrete, and the environmental effects of steam-cured concrete. The specific conclusions are as follows:

(1) Steam curing can accelerate the early reaction process of cement and mineral admixtures, promote the densification of C-(A)-S-H, and promote the development of the early strength of concrete. However, steam curing also causes the degradation of the later strength of concrete. The main reasons are as follows: (1) Thermal damage: During the steam curing process, the surface temperature of the concrete is too high, and the surface pores coarsen due to the expansion of water and steam. (2) Shrinkage cracking: In the heating stage, the concrete expands greatly, and in the subsequent cooling stage, the strength of the concrete cannot resist the shrinkage force, causing cracks to form. (3) DEF: The formation of ettringite is limited during high-temperature steam curing when elements such as S and Al are present in the hydration product; when ettringite forms again in the long-term process, volume expansion causes destruction of the concrete.

(2) It is not advisable to use fly ash as a mineral admixture in steam-cured concrete because it causes insufficient early strength. However, fly ash can be used with highly active admixtures such as slag to ensure that the early strength meets requirements while improving the durability of concrete. New solid waste powder and recycled aggregate, such as limestone powder, metakaolin, and discarded PC, can be used in steam-cured concrete, but traces of harmful substances must be eliminated.

(3) Compared with ordinary concrete, steam-cured PC has higher CO_2_ emissions during the curing process, accounting for approximately 50% of the total emissions. For steam-cured PC, it is necessary to accelerate innovation in energy technologies and promote the replacement of traditional cement with mineral admixtures to minimize CO_2_ emissions.

(4) To alleviate the lack of later strength in steam-cured concrete, it is necessary to ensure adequate curing time of the concrete and use a gentle heating method to give the concrete time to form its initial structure. A heating time of approximately 3 h, and a constant temperature time of approximately 6 h, can ensure the performance of PC and minimize CO_2_ emissions. Additionally, the maximum curing temperature must be kept at approximately 60 °C and should not be too high. If conditions permit, some secondary curing methods, such as soaking in an aqueous solution of lime or standard curing, can be used to alleviate the negative effects of steam curing on concrete. It is necessary to adjust the secondary curing modes according to practical engineering applications.

## Figures and Tables

**Table 1:** Improvement in the early compressive strength of concrete with steam curing compared with standard curing.

Improvement	Age	Type of Concrete	Researchers
47%	1d	100% cement, w/b = 0.45, binder = 360 kg/m^3^	Vaasudevaa [7]
15%	1d	100% cement, w/b = 0.40, binder = 400 kg/m^3^	
92%	1d	100% cement, w/b = 0.36, binder = 474 kg/m^3^	Zhang [8]
31%	1d	100% cement, w/b = 0.35, binder = 450 kg/m^3^	Gesoğlu [9]

**Table 2:** Improvement in the later compressive strength of steam-cured concrete compared with standard-cured concrete.

Improvement	Age	Type of Concrete	Researchers
−20%	28 d	100% cement, w/b = 0.4	Calvo [18]
−6%	28 d	100% cement, w/b = 0.45, binder = 360 kg/m^3^	Vaasudevaa [7]
18%	28 d	100% cement, w/b = 0.40, binder = 400 kg/m^3^	
−11%	90 d	100% cement, w/b = 0.36, binder = 474 kg/m^3^	Zhang [8]
−12%	28 d	100% cement, w/b = 0.35, binder = 450 kg/m^3^	Gesoğlu [9]
−14%	56 d	100% cement, w/b = 0.35, binder = 450 kg/m^3^	

**Table 3:** Harmful emissions per ton of cementitious materials (kg) [53,80,81].

Types	Cement	Fly Ash	Slag
CO_2_	833~944	-	-
SO_2_	0.63	9.1 × 10^−5^	0.2
CO	1.96	9.1 × 10^−3^	0.1
NO_x_	1.79	1.8 × 10^−2^	2.2 × 10^−2^
Particulate matter	0.03	3.2 × 10^−2^	0.1
